# 

**Published:** 1903-03-07

**Authors:** 


					the hospital. "The standard of highest purity."?Lancet, march 7th, 1903
CADBURY'S CADBURY'S
COCOA Mfc JJOl jp  COCOA
ABSOLUTELY PURE. <Pe> ? '? NO DRUGS OR ALKALIES USED
No. 858Tv ol. XXXIII. [aNewS"]
Saturday, March 7th, 1903.
?Su bscriptior^ Ter a, s,] pRICH TWOPENCE.

				

